# Hormone replacement therapy after surgery for stage 1 or 2 cutaneous melanoma

**DOI:** 10.1038/sj.bjc.6601595

**Published:** 2004-02-17

**Authors:** R M MacKie, C A Bray

**Affiliations:** 1 Public Health and Health Policy University of Glasgow, Glasgow G12 8RZ, UK

**Keywords:** malignant melanoma, hormone replacement therapy, prognosis, oestrogen, progestogens

## Abstract

A total of 206 women were followed for a minimum of 5 years after primary melanoma surgery to establish if hormone replacement therapy (HRT) adversely affected prognosis. In all, 123 had no HRT and 22 have died of melanoma; 83 had HRT for varying periods and one has died of melanoma. After controlling for known prognostic factors, we conclude that HRT after melanoma does not adversely affect prognosis.

There is very little literature on the safety of hormone replacement therapy (HRT) for women after diagnosis and apparently successful treatment for melanoma. Two studies, one from Denmark (Osterlind *et al*, 1988) and a recent large study from Sweden (Persson *et al*, 1996), both suggest that HRT is not a risk factor for melanoma development, but no study has been found in the literature suggesting that HRT after melanoma diagnosis affects the prognosis. Despite this, package inserts for HRT warn women that caution should be exercised in taking HRT after melanoma diagnosis. The relevant introductory paragraph on HRT in the British National Formulary freely acknowledges that ‘evidence for caution in melanoma is unsatisfactory and many women may stand to benefit from HRT’ (Joint formulary committee, 2003).

It is therefore not surprising that in melanoma follow-up clinics, a common request from general practitioners is advice on whether or not HRT is contraindicated.

At present in the UK, approximately 3000 women annually will be newly diagnosed with invasive melanoma and, as the average age at diagnosis is in the early 50s (MacKie *et al*, 2002), a high proportion of these women may wish to be considered for HRT therapy.

We therefore undertook this study to provide an evidence base for offering advice to women on the safety or hazard of HRT after surgery for primary melanoma.

## METHODS

The study has full ethical committee approval.

All women in the west of Scotland with melanoma diagnosed between 1990 and 1995, who had been born between 1935 and 1950, were identified from the records of the west of Scotland section of the Scottish Melanoma Group.

Decisions on HRT therapy were made purely by the general practitioner looking after the patient or by the patient's gynaecologist, not by the individual in charge of melanoma follow-up. All patients were followed up for a minimum of 5 years, and the current median follow-up time is 10.6 years.

Melanoma was confirmed in every case and the significant prognostic features of tumour thickness and ulceration were reviewed. Follow-up recorded whether the patient was alive and melanoma free, alive with recurrent melanoma, dead as a result of melanoma or dead of other causes.

### Statistical methods

Univariate analyses for categorical variables were performed using a Chi-squared test of association. Continuous variables were analysed using *t*-tests or Mann–Whitney tests where appropriate. Kaplan–Meier and stepwise Cox regression analyses were used to model the melanoma-free time data uni- and multivariately, respectively.

## RESULTS

**Figure 1:**
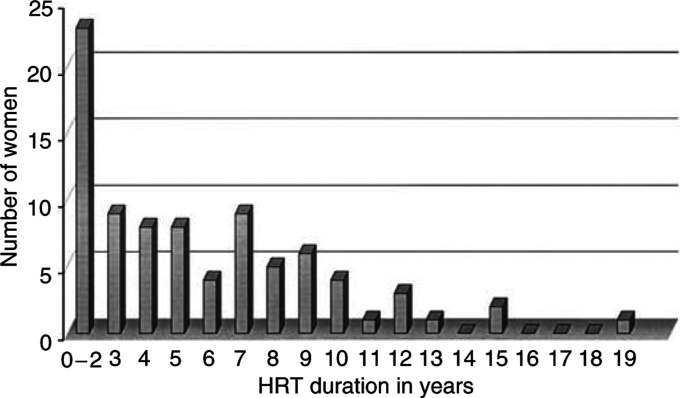
Number of women taking HRT for specified number of years.

A total of 225 women were eligible to enter the study. Information on HRT status and complete melanoma follow-up data were available for 206 (92%) women. In all, 123 women (60%) had received no HRT at any time; 83 received HRT at some time for periods ranging from 1 month to 19 years as shown in Figure 1

. The majority of women received HRT for less than 3 years, mainly for perimenopausal symptoms. A total of 21 women with no uterus had pure oestrogen replacement and 62 had a variety of combined oestrogen/progesterone preparations. One of these women has died of melanoma after having had combined oestrogen/progesterone HRT for 12 months, two are currently alive with recurrent melanoma having had HRT for 7 and 10 years, respectively, and 80 remain alive and melanoma free.

The main prognostic factors for malignant melanoma are tumour thickness and ulceration. Minor factors may be histogenetic type, body site, deprivation category and age. The distributions of these factors for the HRT-positive and HRT-negative groups are shown in Table 1

**Table 1:** Baseline characteristics in HRT^+^ and HRT^−^ cases

	**HRT^+^**	**HRT^−^**	
	***n* (%)**	***n* (%)**	***P*-value**
Ulceration			
Yes	5 (6.2)	21 (17.8)	0.017*
Patients with tumours <1 mm thick	42 (50.6)	58 (47.2)	0.627*
Patients with			
Superficial spreading melanoma	60 (73.2)	84 (69.4)	0.846*
Nodular/polypoid melanoma	15 (18.3)	25 (20.7)	
Lentigo maligna melanoma	4 (4.9)	4 (4.3)	
Acral/mucosal melanoma	1 (1.2)	2 (1.7)	
Other and unspecified melanoma	2 (2.4)	6 (5.0)	
			
Patients with lesions on			
Limbs	59 (72.0)	79 (65.3)	0.602*
Hands and feet	3 (3.7)	6 (5.0)	
Other body parts	20 (24.4)	36 (29.8)	
Deprivation category			
1 and 2	26 (31.7)	32 (26.5)	0.034*
3, 4 and 5	50 (61.0)	64 (52.9)	
6 and 7	6 (7.3)	25 (20.7)	
Age			
Mean	48.65	50.52	0.009**

* *χ*^2^ test of association.

** *t*-test.

. Women in the HRT-positive group were younger and had a lower proportion of ulceration than those in the HRT-negative group. There was no difference in the median tumour thickness in the HRT-positive (0.90 mm) and HRT-negative (1.00 mm) groups (*P*=0.735).

Univariate analysis shows a highly significant survival difference in favour of the HRT-treated group (*P*=0.004). However, the group not offered HRT have a higher proportion of ulcerated primary tumours, recognised as a major prognostic factor (Balch *et al*, 2001a). Once this is controlled for in a multivariate analysis, the significance is reduced but still maintained (*P*=0.007). Tumour thickness was not significantly different between the HRT-positive and -negative groups. The mean age of the HRT-negative group was higher than the HRT-positive group, but when this enters the model the significance of the survival difference is unchanged (*P*=0.007) (Table 2

**Table 2:** Statistical analyses of HRT^+^ and HRT^−^ cases

	**HRT^+^**	**HRT^−^**	
Number of cases	83	123	
Alive and disease free	80	93	
Alive with disease	2	4	
Died of melanoma	1	22	
Died of other causes	0	4	
			
**Cox regression**	**HR (95% CI)**	**HR**	***P*-value**
Unadjusted	0.167 (0.050, 0.555)	1	0.004
Adjusted for ulceration	0.189 (0.057, 0.634)	1	0.007
Adjusted for ulceration and primary tumour thickness	0.136 (0.039, 0.480)	1	0.002
Adjusted for ulceration, primary tumour thickness and age	0.173 (0.048, 0.621)	1	0.007

HR=hazard ratio.

).

Table 3

**Table 3:** Details of patients who have died of melanoma

**Age at melanoma diagnosis**	**Primary tumour thickness (mm)**	**Time from melanoma diagnosis to death**
HRT+ 55	2.0	6 months
HRT− 46	1.6	2 years
HRT− 46	3.1	3 years
HRT−54	1.9	3 years
HRT−57	3.9	1 year
HRT− 55	1.7	1 year
HRT− 44	3.8	6 months
HRT− 46	5.0	2 years
HRT− 55	1.8	2 years
HRT− 55	1.1	10 years
HRT− 56	5.7	2 years
HRT− 55	0.6	6 years
HRT− 55	9.0	1 year
HRT− 59	0.5	3 years
HRT− 46	1.5	4 years
HRT− 59	5.7	1 year
HRT− 49	0.5	7 years
HRT− 48	4.3	10 years
HRT− 56	3.0	1 year
HRT− 51	1.1	7 years
HRT− 45	1.7	4 years
HRT− 50	4.0	1 year

shows the details of all patients dying of melanoma, and illustrates the fact that the range of primary tumour thickness, and time from diagnosis to death is wide.

The final model, based on a stepwise Cox regression, adjusting for ulceration, tumour thickness and age, still showed a survival difference in favour of the HRT-positive group (HR=0.173; 95% CI (0.048, 0.621)).

## DISCUSSION

The object of this study was to prove that HRT therapy was not disadvantageous to women who had received apparently successful surgery for AJCC stage 1 or 2 melanoma. The ideal study to prove this would have been a randomised controlled study, with all women who had had melanoma treated and then wished HRT randomised to receive HRT or placebo. This was not practical in terms of numbers of patients available and willing to give informed consent to be entered into such a study. We therefore carried out this prospective observational study.

Reports from the UK (Ballard, 2002) indicate that 60% of women aged 51–57 years have had some form of HRT, and from the US that 40% of women have had HRT by their 50th birthday (Fletcher and Colditz, 2002). The UK data compared with the 40% of melanoma patients receiving HRT in our study indicate that currently fewer melanoma patients are prescribed HRT than in the general population. It appears that general practitioners and gynaecologists caring for these patients currently select patients in the better prognosis group for HRT as shown by the imbalance in ulcerated melanomas between those who did and did not receive HRT. It is also apparent that the majority of melanoma patients for whom HRT is prescribed receive it for less than 3 years, mainly for perimenopausal symptoms, rather than for long-term prevention of osteoporosis.

Our results show that HRT as used in this cohort of women appears to have no adverse effect on outcome after surgery for localised melanoma and indeed suggests that such therapy may improve prognosis. It is obviously vital to ensure that an imbalance of recognised prognostic factors between the two groups does not explain this unexpected observation. The major currently recognised prognostic factors include tumour thickness and ulceration. As is reported, we found over-representation of women with ulcerated melanomas in the group not receiving HRT, but melanoma thickness was not significantly different between the groups. We have previously reported that women in higher socioeconomic groups have better survival prospects than those in the less-affluent groups (MacKie and Hole, 1996), but again controlling for this did not remove the apparent survival advantage in receiving HRT. Younger women with melanoma have a survival advantage over postmenopausal women (Balch *et al*, 2001b), so it may be that by restoring the endocrine milieu in these women to a premenopausal state, the survival advantage is real.

This study was completed before the results of the WHI (Chlebowski *et al*, 2003), and million women (Million Women Study Collaborators, 2003) studies reported an increased incidence in breast cancers in women receiving HRT of all types, and also an increase in breast cancer mortality in women on HRT in the million women study. These disturbing data will clearly radically alter the current approach to the use of both short- and long-term use of HRT in all women. However, the data on HRT and melanoma obtained in this study indicate that women who have had stage 1 or 2 melanoma successfully treated should be considered for HRT in the same way as those who have never had melanoma.
